# Supplementary material for: Integrative Physiological and Molecular Insights into Drought–Induced Accumulation of Bioactive Compounds in Clinacanthus nutans (Burm.f.) Lindau Leaves
Source: Plants (Basel). 2025 Dec 29;15(1):100. doi: 10.3390/plants15010100 (PMC12787733; doi:10.3390/plants15010100)
Supplement: Supplementary file 1 [file plants-15-00100-s001.zip › plants-4020149-supplementary.pdf]

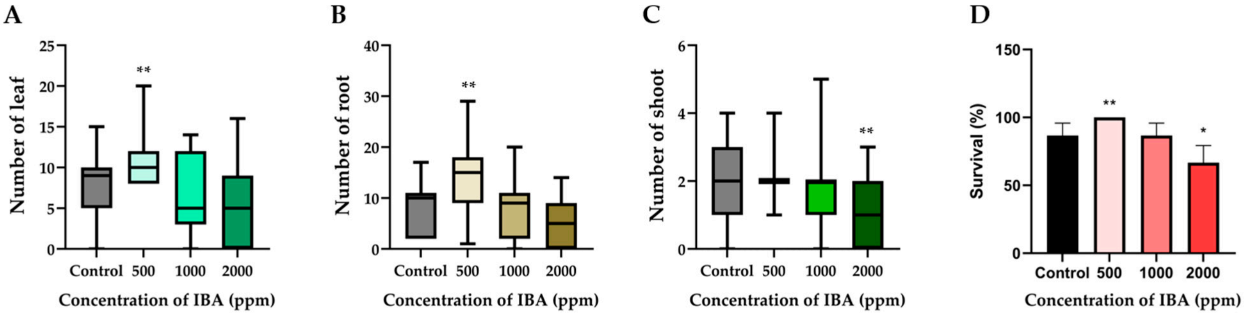

Figure S1. Effects of indole-3-butyric acid (IBA) concentration on the growth of *Clinacanthus nutans* seedlings; evaluated by the number of leaves (A), number of roots (B), number of shoots (C), and survival rate (D) after cutting propagation. Data in panels A–C are presented as box plots, while data in panel D are expressed as mean ± standard error (SE) from fifteen replicates (n = 15). Asterisks (* and **) indicate statistically significant differences compared with the control group at $p < 0.05$ and $p < 0.01$, respectively.

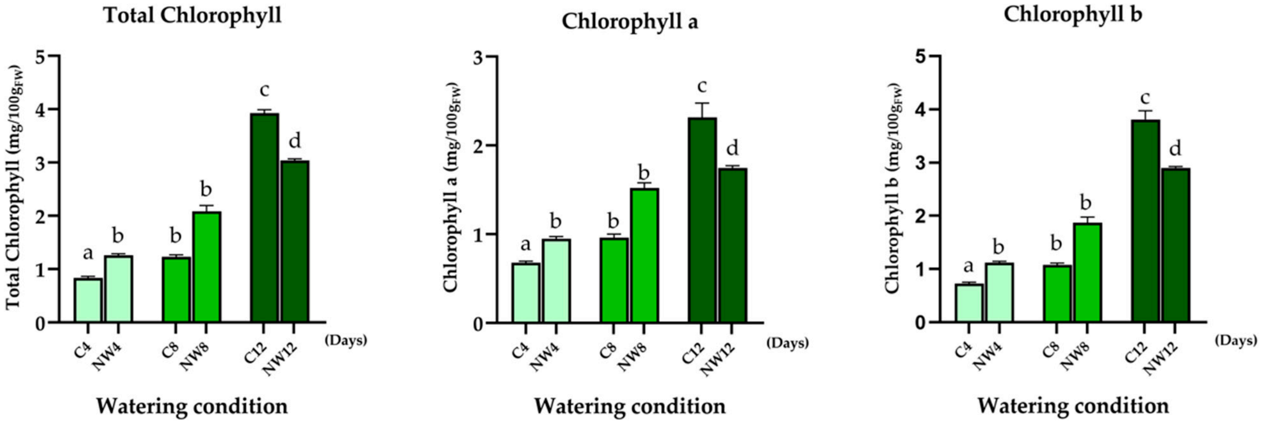

Figure S2. Changes in chlorophyll content in *Clinacanthus nutans* leaves under drought stress; Total chlorophyll content (A), chlorophyll a (B), and chlorophyll b (C) in Clinacanthus nutans leaves under well-watered (normal) and drought-stressed conditions for 4, 8, and 12 days. Data are presented as mean ± standard error (SE) from three replicates (n = 3). Different letters (a–d) indicate statistically significant differences at the 99% confidence level ($p < 0.01$), as determined by Duncan's multiple range test (DMRT).

C

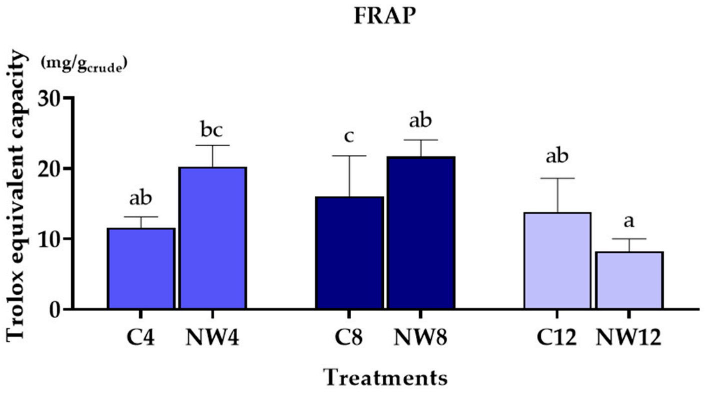

Figure S3. Antioxidant activity of *Clinacanthus nutans* leaf extracts under drought stress. Antioxidant activity of crude extracts from *Clinacanthus nutans* leaves under well-watered (control, C) and drought-stressed (NW) conditions for 4, 8, and 12 days, as evaluated by the FRAP assay. Data are presented from three replicates (n = 3). Different letters (a–e) indicate statistically significant differences at the 99% confidence level ($p < 0.01$).
